# Predicting diabetes-related conditions in need of intervention: Lolland-Falster Health Study, Denmark

**DOI:** 10.1016/j.pmedr.2023.102215

**Published:** 2023-04-20

**Authors:** Søren Lophaven, Neda Esmailzadeh Bruun-Rasmussen, Therese Holmager, Randi Jepsen, Allan Kofoed-Enevoldsen, Elsebeth Lynge

**Affiliations:** a Omicron Aps, Roskilde, Denmark; b Center for Epidemiological Research, Nykøbing Falster Hospital, Denmark; c Steno Diabetes Center Zealand and Department of Endocrinology, Nykøbing Falster Hospital, Denmark

**Keywords:** Diabetes, Risk factors, Health survey, Prediction model, Receiver operating characteristic curve, Screening

## Abstract

In the Danish population, about one-in-ten adults have prediabetes, undiagnosed, poorly or potentially sub-regulated diabetes, for short DMRC. It is important to offer these citizens relevant healthcare intervention. We therefore built a model for prediction of prevalent DMRC.

Data were derived from the Lolland-Falster Health Study undertaken in a rural-provincial area of Denmark with disadvantaged health. We included variables from public registers (age, sex, age, citizenship, marital status, socioeconomic status, residency status); from self-administered questionnaires (smoking status, alcohol use, education, self-rated health, dietary habits, physical activity); and from clinical examinations (body mass index (BMI), pulse rate, blood pressure, waist-to-hip ratio). Data were divided into training/testing datasets for development and testing of the prediction model.

The study included 15,801 adults; of whom 1,575 with DMRC. Statistically significant variables in the final model included age, self-rated health, smoking status, BMI, waist-to-hip ratio, and pulse rate. In the testing dataset this model had an area under the curve (AUC) = 0.77 and a sensitivity of 50% corresponding to a specificity of 84%.

In a health disadvantaged Danish population, presence of prediabetes, undiagnosed, or poorly or potentially sub-regulated diabetes could be predicted from age, self-rated health, smoking status, BMI, waist-to-hip ratio, and pulse rate. Age is known from the Danish personal identification number, self-rated health and smoking status can be obtained from simple questions, and BMI, waist-to-hip ratio, and pulse rate can be measured by any person in health care and potentially by the person him/her-self. Our model might therefore be useful as a screening tool.

## Highlights

• Approximately 10% of adults in Denmark have prediabetes, undiagnosed, or poorly or potentially sub-regulated diabetes.
• The presence of prediabetes, undiagnosed, or poorly or potentially sub-regulated diabetes can be predicted reasonably well.
• The prediction model estimated in this study might be a useful screening tool.

## 1 Introduction

In newer Danish population-based health studies, 8.3%, 11.9%, and 12.4%, respectively, of adult participants had either known diabetes, undiagnosed diabetes or prediabetes as measured from self-reported data on diagnosis and medication, and level of glycated hemoglobin (HbA1c) in blood samples (Jørgensen et al., 2020, Bruun-Rasmussen et al., 2020). While persons with well-regulated diabetes are taken care of, persons with undiagnosed diabetes or prediabetes, as well as persons with poorly controlled or potentially sub-regulated diabetes need intervention from the healthcare system to prevent possible long-term complications. Roughly, 10% of adult Danes suffer from prediabetes, undiagnosed, or poorly controlled/sub-optimally regulated diabetes. As a common term for these conditions, we use “diabetes mellitus related conditions in need of intervention” (DMRC) (Bruun-Rasmussen et al., 2020).

Public health actions focusing on identification, treatment and follow-up are essential for reversing and reducing the human, social and economic consequences of diabetes. This requires easily available information on how to identify potential persons with diabetes at an early stage. There are two types of diabetes prediction models. First, models for identification of risk factors for future incident cases of diabetes; second models for targeted screening for prevalent cases of diabetes (Asgari et al., 2021). Given that we included in our outcome measure both poorly regulated diabetes and prediabetes, our model was in a way a mixture. However, as our focus was on already existing cases of DMRC, our model can best be considered as belonging to the second type of models.

We aimed to determine to what extent data from population registers, self-administered questionnaires and non-invasive clinical assessments can predict presence of DMRC. We used data from the population-based health survey in Lolland-Falster, a rural-provincial part of Denmark with a life expectancy below the national average and with health problems reported more frequently than for the rest of Denmark (Holmager et al., 2021).

## 2 Material and methods

### 2.1 Study population

Data were derived from the Lolland-Falster Health Study (LOFUS), a population-based survey undertaken in a rural-provincial area of Denmark in 2016–2020 (Jepsen et al., 2020). In this study, persons aged 18 years and above were randomly selected from the Central Population Register and invited with their household members of all ages to participate. Invited persons who agreed to participate completed web-based questionnaires prior to a clinical examination and provision of biological samples at one of three study centers. The overall participation rate was 36%. In an earlier study, the DMRC prevalence was investigated for a subset of LOFUS participants (Bruun-Rasmussen et al., 2020). For the present study, we included the entire LOFUS dataset of adult participants aged 20 years and above (n = 15,811) recruited from 2016 to 2020.

### 2.2 Independent variables

#### 2.2.1 Population registers

From the Central Population Register, we used data on sex, age, citizenship, and marital status at the time of participation in LOFUS. Moreover, based on historical address data, LOFUS participants were divided into long-term residents and in-migrants. Long-term residents lived in Lolland-Falster for at least 10 consecutive years prior to the invitation date to LOFUS, while in-migrants had last moved into Lolland-Falster <10 years before the invitation date. From the Socio-Economic Classification (SOCIO13) in Statistics Denmark, we used data on socioeconomic status from 2017. For people of working age (30–64 years), we merged the socioeconomic data into economically self-supported persons and publicly supported persons on transfer income, while those below age 30 or above age 65 years were not classified by socioeconomic status.

#### 2.2.2 Self-administered questionnaires

From the LOFUS questionnaires, we used data on educational levels divided into ‘low’ (≤9 years), ‘medium’ (10–11 years), and ‘high’ (12 + years). Data on frequency of intake of 5+ units of alcohol was based on a single question with four response categories; ‘do not drink’, ‘rarely’, ‘monthly’ and ‘weekly/daily’. Smoking status was based also on a single question with three response categories: ‘current’, ‘former’ and ‘never’. Current self-rated health was measured by one question with four response categories: ‘very good’, ‘good’, ‘fair’ and ‘poor/very poor’. Self-assessment of general dietary habits was obtained from one question and categorized into three responses ‘healthy’, ‘somewhat healthy’ and ‘unhealthy’. Leisure time physical activity during the past 12 months was measured by one question with three response categories: 1) ‘low’, mainly sedentary activities (TV-watching, reading), 2) ‘moderate’, light physical activities ≥ 4 h per week (walking, bicycling, light gardening), 3) “high”, sports or other more vigorous activities ≥ 4 h per week (heavy gardening) or vigorous physical activity several times per week (heavy exercise or competitive sports).

#### 2.2.3 Non-invasive clinical assessments

Data from clinical assessments included body mass index (BMI) based on measured height and weight at the clinical examination and calculated as weight in kilogram divided by height in meters squared (kg/m^2^), and for descriptive purposes categorized into ‘underweight’ (<18.5), ‘normal’ (18.5–24.9), ‘overweight’ (25.0–29.9), and ‘obese’ (≥30.0). Systolic and diastolic blood pressure was measured and categorized into ‘normal’, ‘high normal’ and hypertension ‘grade 1’, ‘2’, and ‘3’ (Dansk Kardiologisk Selskab). Pulse rate was measured with an oximeter in beats per minute. Waist-hip ratio (WHR) was calculated by waist-circumference divided by hip circumference. For details see Supplementary Table 1.

### 2.3 Outcome variable

Non-fasting blood samples were collected at the three LOFUS study centers and analyzed at the Department of Clinical Biochemistry at Nykøbing Falster Hospital accredited by the standard ISO 15189. We used data on HbA1c. For participants with self-reported diabetes and/or self-reported use of antidiabetic medication including insulin and other antidiabetic medications, well-controlled diabetes was defined as those with HbA1c < 48 mmol/mol (<6.5%); potentially sub-regulated diabetes as those with HbA1c 48–59 mmol/mol (6.5–7.5%), and poorly controlled diabetes as those with HbA1c ≥ 60 mmol/mol (≥7.6%). For participants without self-reported diabetes and/or use of antidiabetic medication, no diabetes was defined as HbA1c < 42 mmol/mol (<6.0%); prediabetes as HbA1c 42–47 mmol/mol (6.0–6.5%); and undiagnosed diabetes as HbA1c ≥ 48 mmol/mol (≥6.5%) (Bruun-Rasmussen et al., 2020). It was not possible to distinguish between type 1 and type 2 diabetes. Participants with missing data on HbA1c, self-reported diagnosis of diabetes, and/or self-reported use of antidiabetic medication were labeled ‘missing’.

### 2.4 Statistical analysis

We tabulated summary statistics of the data described above by diabetes-related diagnostic groups. Furthermore, as the purpose of the study was identification of predictors for DMRC, participants with prediabetes, or undiagnosed, poorly controlled, or potentially sub-controlled diabetes were merged into one group designated DMRC, and participants with no diabetes, well-regulated diabetes, or missing were collapsed into one comparison group.

In the analysis, sex, citizenship, marital status, migration status, socio-economic status, education, alcohol consumption, smoking status, self-rated health, dietary habits, physical activity and blood pressure were treated as categorical variables, while age, BMI, pulse rate and waist-to-hip ratio were treated as continuous variables. We performed univariate logistic regression with one explanatory variable at a time. We tested each explanatory variable for significance on a 5% level using likelihood-ratio tests. Explanatory variables found to be significantly associated with the binary response variable were included for further predictive analysis using a multiple logistic regression model.

The full LOFUS dataset of 15,811 participants was afterwards randomly split into a training dataset consisting of 70% of the participants and a test dataset with the remaining 30%. A full multiple logistic regression model with the candidate set of explanatory variables identified from the univariate analyses described above was run on the training dataset. The full model was reduced to include only statistically, significantly explanatory variables using a stepwise regression approach. The full and reduced models estimated on the training dataset were used to calculate predictions for the test dataset and a receiver operating characteristic curve was plotted to illustrate the results and the Area Under the Curve (AUC) was calculated.

The statistical analysis approach described above was applied to four sets of predictor variables:

- Model 1: Variables from population registers;
- Model 2: Variables available in population registers and self-administered questionnaires;
- Model 3: Variables available in population registers, self-administered questionnaires and clinical assessment;
- Model 4: Variables from model 3 with p-values < 0.001.

As a sensitivity analysis we divided the DMRC group into those with poorly- and potentially sub-controlled diabetes as one group, and those with prediabetes and undiagnosed diabetes as another group. The rational being that the first group was expected already to be in contact with the health care system concerning their diabetes, while this was not expected for the second group. Furthermore, we performed an additional sensitivity analysis in which individuals with missing information on diabetes status were excluded. All statistical analyses were performed in R ver. 4.1.0 (R Core Team, 2020). The analysis took place at the research server in Statistics Denmark.

### 2.5 Ethics approval and consent to participate

Participants provided written informed consent and the Region Zealand’s Ethical Committee on Health Research (SJ-421) and the Danish Data Protection Agency (REG-24-2015) approved the study. In the case of abnormal laboratory results, the LOFUS participant was informed in a return of results-letter and advised to consult his/her general practitioner. All participants were able to check the results of their biochemical analyses on their electronic health records on https://www.sundhed.dk.

## 3 Results

In total, 10% (n = 1575) of the adult LOFUS participants had DMRC, and by far the majority, 90% (n = 14236) of LOFUS participants in the comparison group had no diabetes, Table 1.

**Table 1:** Number of LOFUS participants by variables from population registers and diabetes-related status.

	(1) Normal	(2) Well regulated diabetes	(3) Pot. sub-regulated diabetes	(4) Poorly Controlled diabetes	(5) Pre-diabetes	(6) Undiagnosed diabetes	(7) Missing	(8) =non-DMRC(1), (2) , (7)	(9) = DMRC(3), (4), (5), (6)	(10) Total	(11) DMRC/ Total
	n	n	n	n	n	n	n	n	n	n	%
**Total**	12,798	330	341	170	927	128	1108	14,236	1575	15,811	10.0
**Sex**											
Men	5821	190	211	118	450	79	562	6573	858	7431	11.5
Women	6977	140	130	61	477	49	546	7663	717	8380	8.9

**Citizenship**											
Denmark	12,511	326	340	177	907	127	988	13,825	1551	15,376	10.1
Other	287	4	1	2	20	1	120	411	24	435	5.5

**Age in years**											
Mean	56.3	67.1	66.7	63.7	65.7	64.0	53.9	56.4	65.5	57.3	
Median	57.7	68.3	67.7	64.7	66.7	64.6	53.2	57.8	66.7	59.0	

**Age in years**											
20–39	2039	6	2	6	18	1	279	2324	27	2351	1.1
40–59	5055	55	80	52	247	43	395	5505	422	5927	7.1
60–79	5200	243	241	115	592	77	356	5799	1025	6824	15.0
80+	504	26	18	6	70	7	78	608	101	709	14.2

**Marital status**											
Divorced/single/widow	4035	106	104	59	276	43	119	4260	482	4742	10.2
Married/cohabitant	8652	219	236	118	640	85	196	9067	1079	10,146	10.6
Missing	111	5	1	2	11	0	793	909	14	923	

**SOCIO13**											
Age < 30 years	861	3	0	3	1	1	128	992	5	997	0.01
Self-support	6516	71	89	50	306	41	421	7008	486	7494	6.5
Public support	1312	51	46	39	96	24	210	1573	205	1778	11.5
Age ≥ 65 years	4103	205	206	87	523	62	344	4652	878	5530	15.9
Missing	6	0	0	0	1	0	5	11	1	12	

**Residency status**											
Long-term residency	10,983	277	304	154	822	107	841	12,101	1387	13,488	10.3
In-migrant	1802	52	36	25	104	21	266	2120	186	2306	8.1
Missing	13	1	1	0	1	0	1	15	2	17	

Pot. = potentially.

DMRC = diabetes mellitus related condition in need of intervention.

SOCIO13 = persons aged 30–64 years earning their own income (self-support) or on public transfer income (public support).

Long-term resident = lived in Lolland-Falster for at least 10 years before LOFUS invitation date.

In-migrant = moved to Lolland-Falster within the last 10 years before LOFUS invitation date.

n: Number of participants.

%: Percent DMRC out of total. Percent were not calculated for the ‘Missing’ category or for continuous variables.

The proportion with DMRC was slightly higher in men, 11.5%, than in women 8.9%, and it was higher in Danish citizens, 10.1%, than in citizens of other countries, 5.5%. As expected, DMRC was most frequent in LOFUS participants above the age of 60 years, 14–15%, and the DMRC proportion was more than doubled among 30–64 years old, publicly supported persons, 11.5%, compared with 30–64 years old, self-supported persons, 6.5%.

Current and former smokers included higher proportions of persons with DMRC, 12–13%, than never smokers, 8%, Table 2.

**Table 2:** Number of LOFUS participants by variables from LOFUS questionnaire and diabetes-related status.

	(1) Normal	(2) Well regulated diabetes	(3) Pot. sub- regulated diabetes	(4) Poorly Controlled diabetes	(5) Pre-diabetes	(6) Undiag- nosed diabetes	(7) Missing	(8) =non-DMRC(1), (2) , (7)	(9) = DMRC(3), (4) , (5), (6)	(10) Total	(11) DMRC/ Total
	n	n	n	n	n	n	n	n	n	n	%
**Total**	12,798	330	341	170	927	128	1108	14,236	1575	15,811	

**Smoking status**											
Current	2410	63	59	40	213	25	76	2549	337	2886	11.7
Former	4391	156	157	76	379	51	111	4658	663	5321	12.5
Never	5960	109	123	63	327	52	132	6201	565	6766	8.4
Missing	37	2	2	0	8	0	789	828	10	838	

**Alcohol use**											
Does not drink	2415	65	75	42	201	16	68	2548	334	2882	11.6
Rarely	6463	133	147	74	403	57	129	6725	681	7406	9.2
Monthly	1698	28	35	12	98	17	37	1763	162	1925	8.4
Weekly/daily	967	36	30	8	61	13	28	1031	112	1143	9.8
Missing	1255	68	54	43	164	25	846	2169	286	2455	

**Education in years**											
Low (≤9)	4627	191	193	100	456	68	168	4986	817	5803	14.1
Medium (10–11)	4253	95	97	46	319	35	74	4422	497	4919	10.1
High (≥12)	3805	39	48	28	136	25	69	3913	237	4150	5.7
Missing	113	5	3	5	16	0	797	915	24	939	

**Self-reported health**											
Very good	1632	17	11	7	76	11	22	1671	105	1776	5.9
Good	7463	134	137	56	457	59	163	7760	709	8469	8.4
Fair	3172	152	152	92	324	47	127	3451	615	4066	15.1
Poor/very poor	510	26	40	24	66	11	28	564	141	705	20.0
Missing	21	1	1	0	4	0	768	790	5	795	

**Dietary habit**											
Very healthy/healthy	6104	149	150	72	418	41	146	6399	681	7080	9.6
Somewhat healthy	6013	161	175	93	468	76	144	6318	812	7130	11.4
Unhealthy/very unhealthy	651	18	14	13	38	11	29	698	76	744	9.8
Missing	30	2	2	1	3	0	789	821	6	827	

**Physical activity**											
Low (mainly sedentary)	1400	66	71	42	144	27	60	1526	284	1810	15.7
Moderate (light ≥ 4 h per week)	7776	198	205	103	621	85	190	8164	1014	9178	11.0
High (sport etc. ≥ 4 h per week)	3543	58	60	33	150	15	64	3665	258	2923	6.6
Missing	79	8	5	1	12	1	794	881	19	900	

In the LOFUS dataset, data on alcohol consumption were missing for 16% of participants. Amongst the consumption groups, DMRC was most common in non-drinkers, 11.6%. The proportion of persons with DMRC varied from 5.7% in those with 12+ years of schooling to 14.1% among those with 9 years or less of schooling. There was a steep gradient in the proportion of participants with DMRC across self-rated health with 5.9% in those reporting very good health to 20% in those reporting poor/very poor health. A steep gradient was seen for proportion of participants with DMRC across physical activity group from 15.7% in those with low activity to 6.6% in those with high activity.

A steep gradient was also seen for proportion of participants with DMRC across BMI-groups from 3.3% in underweight persons to 18.2% in obese persons, Table 3. For hypertension, the DMRC proportion was 6.0% for those with normal blood pressure to 12–13% among those with different grades of hypertension.

**Table 3:** Number of LOFUS participants by variables from clinical examination and diabetes-related status.

	(1) Normal	(2) Well regulated diabetes	(3) Pot. sub-Regulated diabetes	(4) Poorly controlled diabetes	(5) Pre-diabetes	(6) Undiag- nosed diabetes	(7) Missing	(8) =non-DMRC(1), (2) , (7)	(9) = DMRC(3), (4) , (5), (6)	(10) Total	(11) DMRC/ Total
	n	n	n	n	n	n	n	n	n	n	%
**Total**	12,798	330	341	170	927	128	1108	14,236	1575	15,811	

**Body Mass Index (kg/m^2^)**											
Mean	26.9	30.1	31.2	30.7	29.7	32.3	27.7	27.0	30.3	27.4	
Medium	26.2	29.3	30.6	29.4	29.1	31.8	27.0	26.4	29.7	26.7	
Missing (n)	251	15	16	7	25	3	55	321	51	372	

**Body Mass Index** **(kg/m^2^)**											
Underweight (<18.5)	161	0	1	0	5	0	17	178	6	184	3.3
Normal (18.5–24.9)	4661	48	33	25	157	11	322	5031	226	5257	4.3
Overweight (25.0–29.9)	4880	127	114	66	345	35	419	5426	560	5986	9.4
Obese (≥30)	2845	140	177	81	395	79	295	32,808	732	4012	18.2
Missing	251	15	16	7	25	3	55	321	51	372	

**Blood pressure**											
Normal	4339	67	66	27	195	23	463	4869	311	5180	6.0
High normal	2853	64	78	47	213	20	200	3117	358	3475	10.3
Hypertension Grade 1	3621	124	139	70	332	43	296	4041	584	4625	12.6
Hypertension Grade 2	1555	59	51	30	142	37	113	1727	260	1987	13.1
Hypertension Grade 3	284	11	5	2	33	2	18	313	42	355	11.8
Missing	146	5	2	3	12	3	18	169	20	189	

**Pulse (beats/minute)**											
Mean	66.7	67.7	70.4	72.2	69.0	71.8	69.3	66.9	69.9	67.2	
Median	66.0	67.0	70.0	72.5	69.0	70.0	68.0	66.0	69.0	66.0	
Missing (n)	52	4	5	1	4	0	3	59	10	69	

**Waist-to-hip ratio**											
Mean	0.9	1.0	1.0	1.0	1.0	1.0	0.9	0.9	1.0	0.9	
Median	0.9	1.0	1.0	1.0	1.0	1.0	0.9	0.9	1.0	0.9	
Missing (n)	131	5	5	1	15	4	18	154	25	179	

In the training dataset, the univariate logistic regression models with one explanatory variable at a time, all explanatory variables, except marital status, were found to be statistically significant, and consequently all but one of the variables described above were included in the multiple logistic regression model developed on the training dataset. Stepwise regression was applied to reduce the model to include only statistically significantly explanatory variables. In model 1, age, SOCIO13 and sex were statistically significant variables, Table 4, see also Supplementary Table 1. In model 2; age, self-rated health, education, physical activity, SOCIO13, sex and dietary habits, were statistically significant variables. In model 3, BMI, age, waist-to-hip ratio, self-rated health, pulse rate and smoking status were statistically significant variables. In model 4, we kept only the variables from model 3 with p-values < 0.001.

**Table 4:** P-values for the four multiple regression models when applied on the training set.

**Predictor**	**p-value (model 1)**	**p-value (model 2)**^2^	**p-value (model 3)**^3^	**p-value (model 4)**^4^
Age (continuous)	3.165e-20	1.278e-15	2.088e-19	1.742e-65
SOCIO13	1.826e-9	0.0212	0.360	Not part of model 4
Sex	2.747e-7	1.487e-4	0.568	Not part of model 4
Residency status	0.452	0.147	0.534	Not part of model 4
Citizenship	0.480	0.488	0.487	Not part of model 4
Self-rated health	Not part of model 1	3.260e-13	3.435–8	4.805e-8
Physical activity	Not part of model 1	0.000378	0.0634	Not part of model 4
Dietary habit	Not part of model 1	0.00803	0.959	Not part of model 4
Education	Not part of model 1	0.0437	0.0206	Not part of model 4
Alcohol use	Not part of model 1	0.561	0.396	Not part of model 4
Smoking status	Not part of model 1	0.891	6.498e-4	4.331e-5
BMI (continuous)	Not part of model 1	Not part of model 2	2.265e-30	3.663e-33
Waist to hip ratio (continuous)	Not part of model 1	Not part of model 2	1.570e-11	1.911e-25
Pulse rate (continuous)	Not part of model 1	Not part of model 2	6.147e-8	6.508e-6
Blood pressure	Not part of model 1	Not part of model 2	0.338	Not part of model 4
Marital status	P = 0.3817317 in univariate model	P = 0.3817317 in univariate model	P = 0.3817317 in univariate model	Not part of model 4

^1^ : Model with predictor variables from health registers.

2 : Model with predictor variables from health registers and questionnaires.

3 : Model with predictor variables from health registers, questionnaires and clinical assessments.

4 : Model with the variables from the model above with p-values < 0.001 (health registers, questionnaires and clinical assessments).

**Fig. 1:**
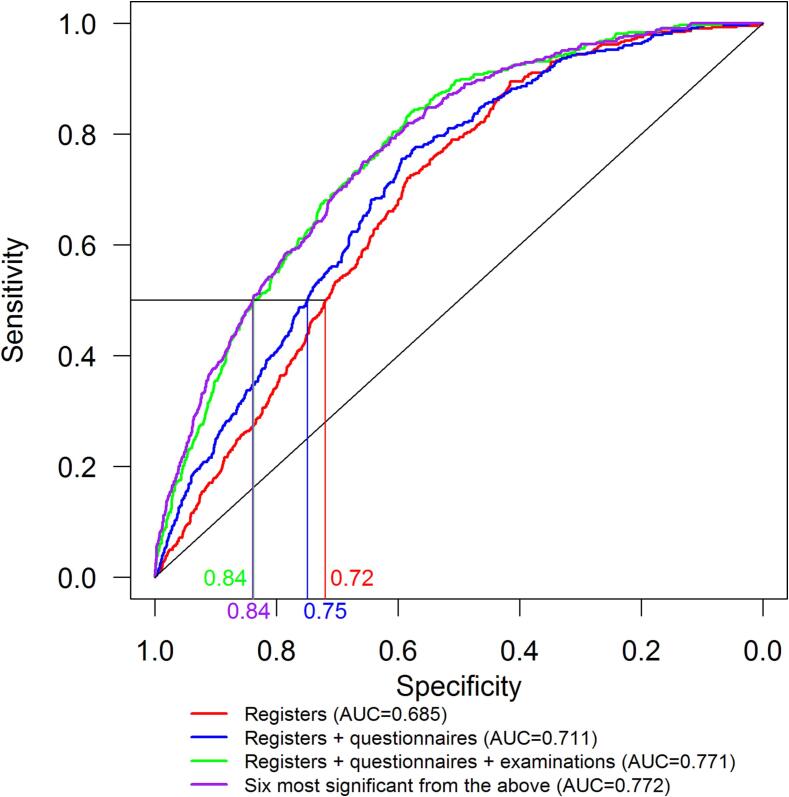
.

For model 1, the AUC for the test dataset was AUC = 0.685; for model 2 it was AUC = 0.711, and for model 3 and 4 it was AUC = 0.771 and 0.772, respectively. A sensitivity of 50%, resulted for model 1 in a specificity of 72%; for model 2 in a specificity of 75%; for models 3 and 4 in a specificity of 84%, Fig. 1 and Supplementary Figures S1, S2, S3 and S4. All the sensitivity analyses performed showed similar results (data not shown).

## 4 Discussion

### 4.1 Main findings

People with prevalent prediabetes or undiagnosed, poorly controlled or potentially sub-regulated diabetes need intervention from the health care system to prevent possible long-term complications. In a population, where this group of people constituted about 10% of the adult population, we found an AUC of almost 0.77 for the prediction of prevalent DMRC with a model based on six easily obtainable characteristics; age, self-rated health, smoking status, and measured BMI, waist-to-hip ratio, and pulse rate.

### 4.2 Other studies

In a systematic review of newer prediction models for type 2 diabetes in the general population, Asgari et al (Asgari et al., 2021) identified 22 models for the detection of undiagnosed diabetes. As risk factors, all 22 models, apart from one, included age; family history of diabetes in 15 models, hypertension/blood pressure and waist-to-hip ratio in 14 models; BMI in 12 models; sex in 10 models, and other risk factors more rarely. The median (interquartile range) AUC was 0.77 (95% CI 0.74–0.81). There is a notable overlap between the risk factors included in the models review by Asgari et al. and the risk factors included in our final model, as we had also age, sex, waist-to-hip ratio, and BMI. Blood pressure was tested out of our final model 3, while pulse rate remained, and we did not have data on family history of diabetes. Nevertheless, the AUC of our model 4 reached the same AUC of 0.77 as the median of the 22 models reviewed by Asgari et al, of which 15 included family history of diabetes.

Only two of the models reviewed by Asgari et al. used European data. Stiglic et al. (Štiglic et al., 2018) developed a model for Slovenia reaching an AUC = 0.85, but this model was one of the few including biomarker data in the form of history of high blood glucose. Gray et al. (Gray et al., 2013) developed a model for Portugal including only age, sex, BMI and current hypertension as predictors. An AUC = 0.74 was found for prevalent type 2 diabetes.

One previous Danish model (Glümer et al., 2004) resembled ours as the purpose was detection of prevalent, unknown diabetes. The model built on the Inter99 study with data collected in the south-western Copenhagen County from 1999 to 2001. Half of the data were used for model training, and the other half for testing. Age, sex, BMI, hypertension, physical activity and parents’ history of diabetes were predictors in the final model, AUC = 0.76 in the testing dataset, and AUC = 0.80 in an independent dataset collected via general practitioners in 1999 in Aarhus, Denmark (Glümer et al., 2004).

### 4.3 Strengths and limitations

Our study population derived from a population-based health survey. Data came from public registers, self-administered questionnaires and clinical non-invasive examinations. We included the three sets of data stepwise in the analysis. In this process, some variables changed status, e.g., sex was a highly, statistically significant variable when only register- and questionnaire data were included in the analysis, but sex became statistically insignificant when also clinical data were taken into account, reflecting difference between men and women in the clinical data.

We did not have data on family history of diabetes, the most widely used variable in other models for prediction of prevalent diabetes. We had data on blood pressure, another widely used variable, but in our model this variable was statistically non-significant in the multivariate analysis. Nevertheless, the AUC = 0.77 in our model was at the level of the average AUC = 0.77 found by Asgari et al. (Asgari et al., 2021) for models predicting prevalent diabetes, and at the level of AUC = 0.76 to 0.81 found by Kengne et al. (Abbasi et al., 2012) for models predicting incident diabetes based on European data.

A potential limitation at the population-basis for applicability of our model is the fact that only 36% of the invited persons participated in the health survey. It should though be taken into account that when selectivity in participation was investigated half-way through the survey (Jepsen et al., 2020), only a moderate gradient was seen in non-participation by e.g. education; relative risks from 1 in high, to 1.13 in medium, to 1.38 in low. However, when this gradient is combined with the gradient in prevalence of DMRC across education among the study participants, we may anticipate that the true prevalence of DMRC in the Lolland-Falster population is higher than the recorded 10%.

### 4.4 Clinical implications

In investigating performance of 12 models for prediction of incident diabetes in datasets from eight European countries, Kengne et al. concluded that “model performance differed across countries and no model outperformed the others enough to be uniquely recommended”. This finding seems very relevant also when it comes to models for prediction of prevalent diabetes or diabetes-related conditions. Our model based on data from Lolland-Falster, a rural-provincial, health disadvantaged area of Denmark, reached an AUC = 0.77 for prediction of prevalent DMRC. However, our model did not include family history of diabetes; the most widely used predictor in other models (Asgari et al., 2021), and the lack of this variable might be a limitation for a wider applicability. Nevertheless, besides locally the model might be useful in similar settings where data are not available for construction of local models.

Our model is very user-friendly. In Denmark, age, in terms of date of birth, is included in the personal identification numbers used universally in healthcare. Data on self-rated health and smoking status can be obtained from very simple questions with 3–4 response categories. BMI, waist-to-hip ratio and pulse rate can be measured by any person in health care and potentially by the person him/her-self. The model could form the basis for screening for prevalent DMRC. Exactly where to set the possible cut-off points for the model variables in a screening program depends on the chosen benefit-to-harm ratio. In the model, a sensitivity of 50% corresponded to a specificity of 84%. This means that at the cut-off points, where half of the prevalent DMRC cases will be detected, 16% of those identified as possible cases will not have DMRC. In this potential DMRC-screening, participants testing positive will be recommended to have a blood-sample taken for measurement of HbA1c. For participants with HbA1c levels < 42 mmol/mol (<6.0%), their screening test will be false positive, and no further action will be taken. This might be an acceptable harm to pay.

## 5 Conclusions

Based on health survey data from a rural-provincial part of Denmark, we found that the presence of diabetes mellitus related conditions in need of intervention could be predicted from age, self-rated health, smoking status, BMI, waist-to-hip ratio and pulse rate. In this simple model, an AUC of 0.77 was found, which is in line with AUC-values of diabetes prediction models including data on family history of diabetes. As found for other diabetes prediction models, our model might not be generally applicable but useful in the local and similar settings.

## Declaration of Competing Interest

The authors declare that they have no known competing financial interests or personal relationships that could have appeared to influence the work reported in this paper.

## Data Availability

Data will be made available on request.
