# Supplementary material for: Predicting diabetes-related conditions in need of intervention: Lolland-Falster Health Study, Denmark
Source: Prev Med Rep. 2023 Apr 20;33:102215. doi: 10.1016/j.pmedr.2023.102215 (PMC10201856; doi:10.1016/j.pmedr.2023.102215)
Supplement: Supplementary data 1 [file mmc1.docx]

March 29, 2023

**Lophaven et al: Predicting diabetes-related conditions in need of intervention: Lolland-Falster Health Study, Denmark**

**Supplementary material**


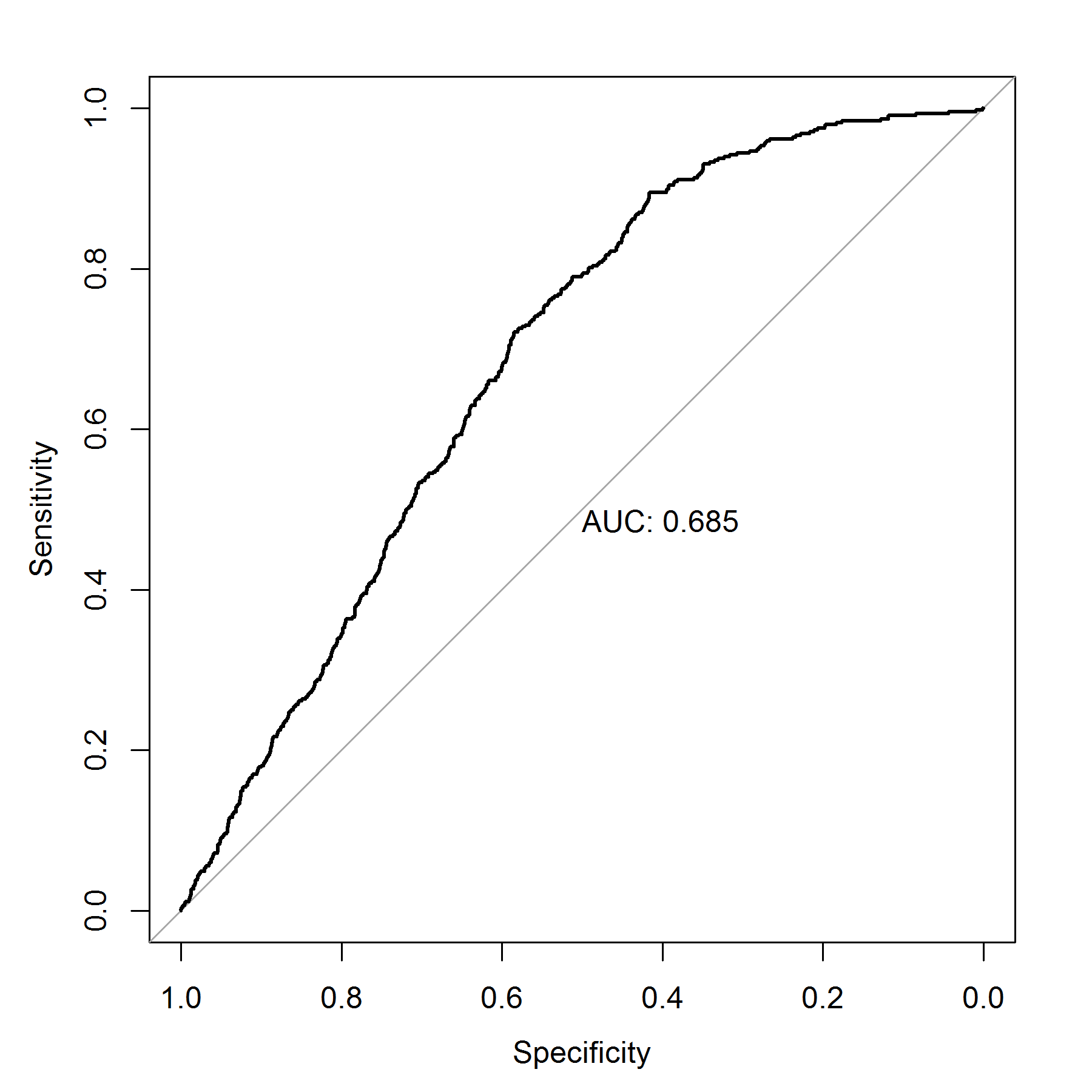


Figure S1: Receiver Operating Characteristic curve for the predicting DM-related conditions in need of intervention. The model includes variables available in Danish health registers


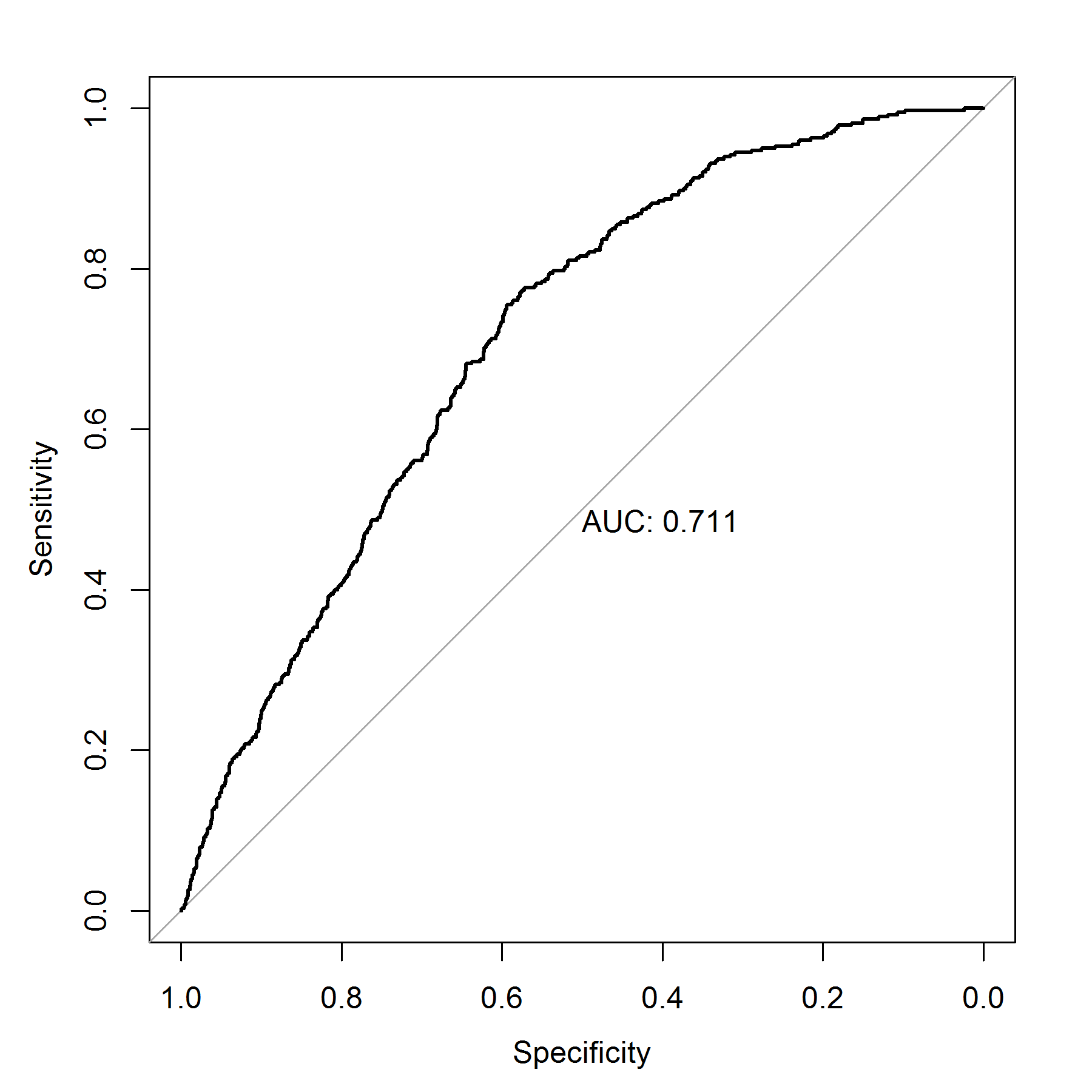


Figure S2: Receiver Operating Characteristic curve for the predicting DM-related conditions in need of intervention. The model includes variables available in Danish health registers and selected questionnaire data from LOFUS


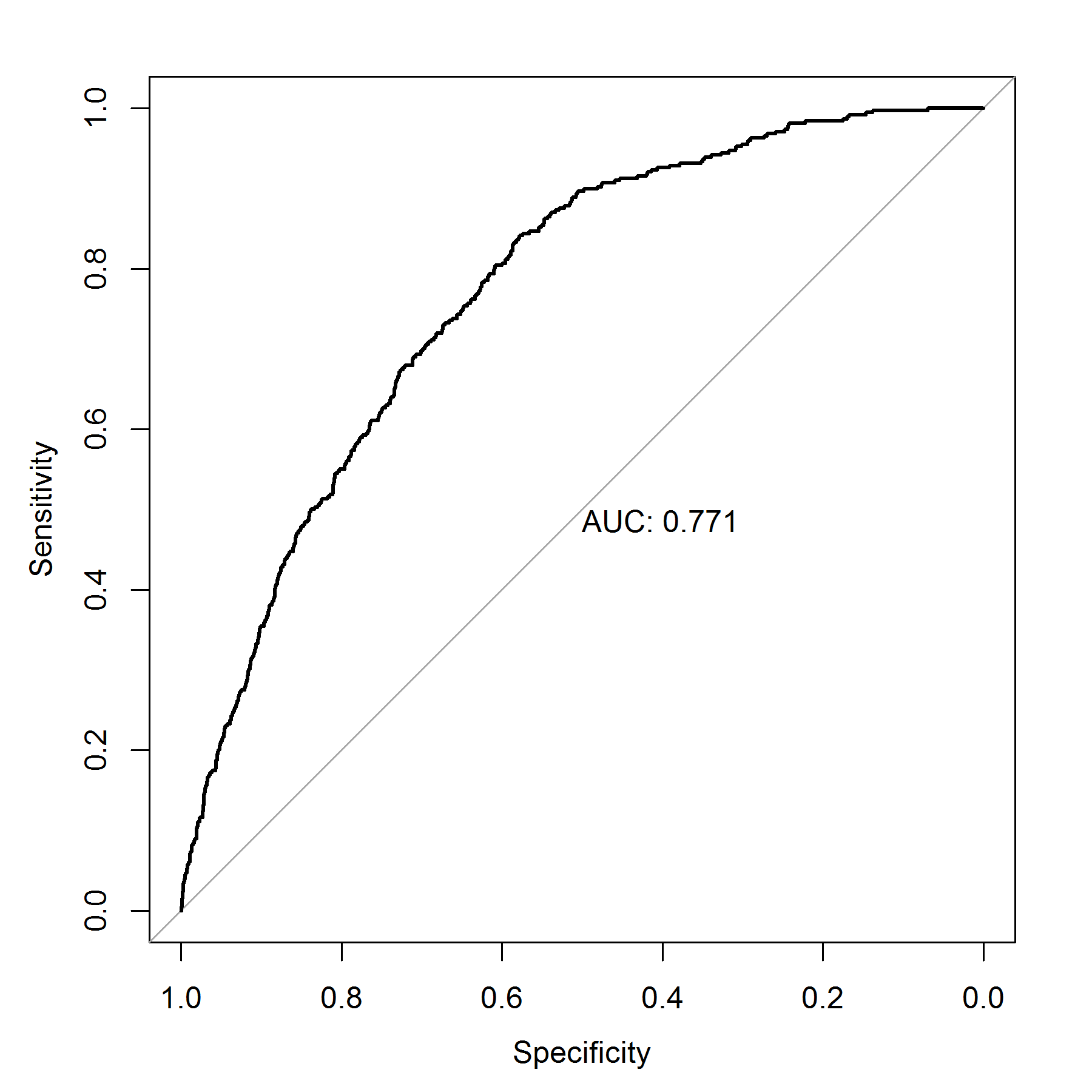


Figure S3: Receiver Operating Characteristic curve for the predicting DM-related conditions in need of intervention. The model includes variables available in Danish health registers as well as selected questionnaire and clinical assessment data from LOFUS


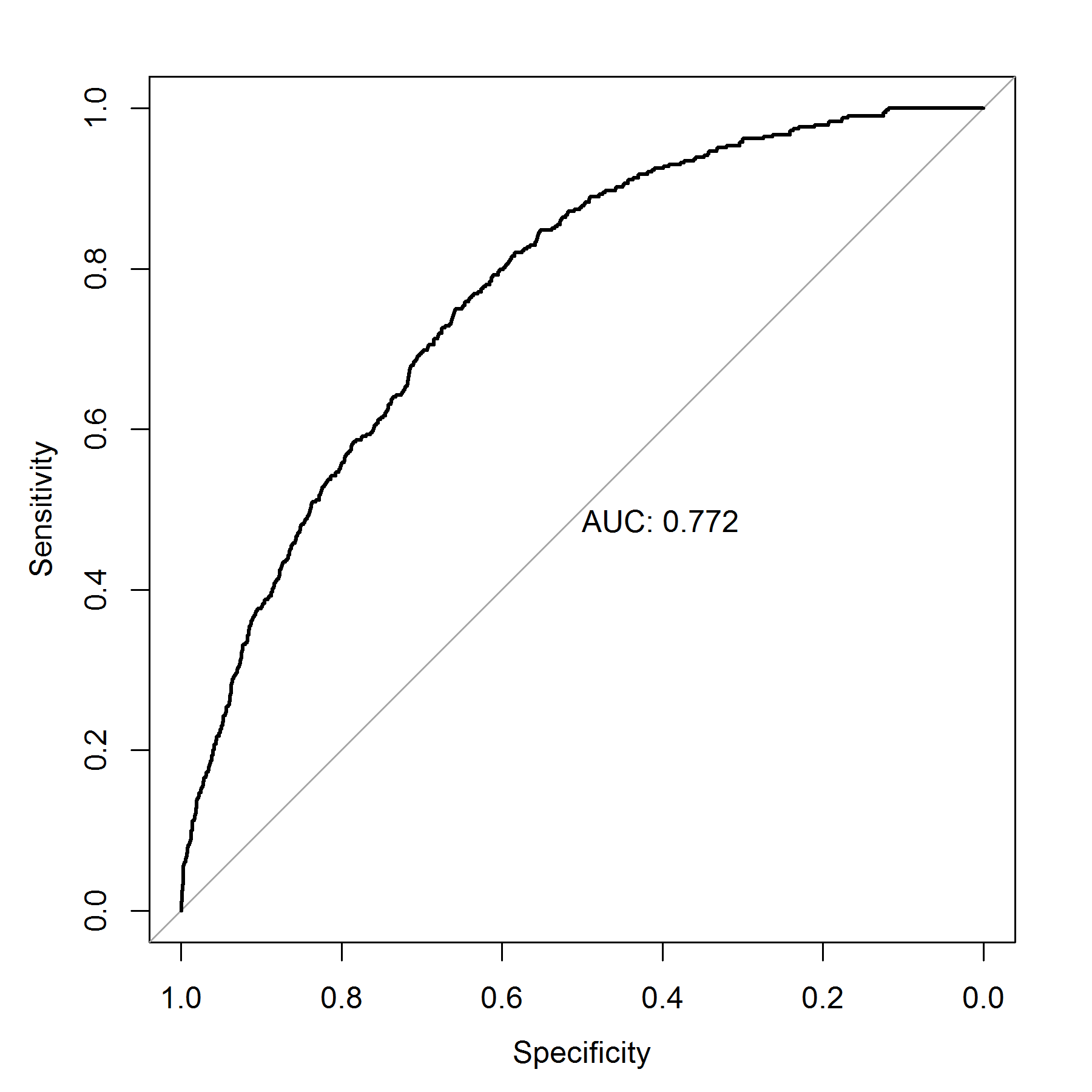


Figure S4: Receiver Operating Characteristic curve for the predicting DM-related conditions in need of intervention. The model includes the seven most significant variables from the model above (Danish health registers as well as selected questionnaire and clinical assessment data from LOFUS)

| Height | Height without shoes was measured in the standing position using the SECA 216 wall-mounted stadiometer. |
| --- | --- |
| Weight | Weight was measured in the non-fasting state, barefoot, and with light clothes using either the Tanita WB-110A digital medical scale, the Tanita Body Composition Analyzer BC-420MA III, or the Tanita Body Composition Analyzer DC-430MA. Clothes weight of one kg was subtracted. |
| Body Mass Index | Body mass index was based in measured height and weight at the clinical examination and calculated as weight in kilograms divided by height in meters squared (kg/m^2^), and for descriptive purposes categorized into ‘underweight’ (<18.5), ‘normal’ (18.5-24.9), ‘overweight’ (25.0-29.9), and ‘obese’ (≥30.0). |
| Blood pressure | Systolic and diastolic blood pressures were measured after five minutes of rest and based on three consecutive digital measurements on the upper left arm (apparatus type Welch Allyn Connex pro BPO 3400). The mean values of the second and third measurements were used in this study (only one measurement was used if the other was missing). For classification of blood pressure, see [12] Supplementary TableS3. |
| Pulse rate | Pulse rate was measured in beats per minute once in the left index finger in the supine position after five minutes of resting using the Nellcor Portable SpO2 Patient Monitoring System, PM10N. |
| Waist-hip ratio (WHR) | Waist- and hip- circumference were measured to the nearest centimeter in the standing position with unelastic measurement tape. The skin was covered by a thin layer of clothing during measurement. Waist-to-hip-ratio was calculated as waist-circumference divided by hip circumference. |

Table S1. Definition of biological exposure variables
